# Infrared light field imaging system free of fixed-pattern noise

**DOI:** 10.1038/s41598-017-13595-7

**Published:** 2017-10-12

**Authors:** Pablo A. Coelho, Jorge E. Tapia, Francisco Pérez, Sergio N. Torres, Carlos Saavedra

**Affiliations:** 1 Departamento de Ingeniería Eléctrica, Universidad de Concepción, Casilla, 160-C Concepción, Chile; 2 Center for Optics and Photonics, Universidad de Concepción, Casilla, 4012 Concepción, Chile; 3 Departamento de Física, Universidad de Concepción, Casilla, 160-C Concepción, Chile

## Abstract

Digital photonic sensors have greatly evolved to maximize sensitivity and spatial, spectral, and temporal imaging resolution. For low-energy photons, new designs have generated new types of noise that degrade the formed-image signal-to-noise ratio to values lower than 1. Fixed-pattern noise (FPN), which is produced by the non-uniform focal-plane-array optoelectronics response, is an ill-posed problem in infrared and hyperspectral imaging science. Here, we experimentally show that the FPN behaves as an object at a depth of infinity when a light field is captured by an imaging system. The proposed method is based on the capture of the light field of a scene and digital refocusing to any nearby objects in the scene. Unlike standard techniques for FPN reduction, our method does not require knowledge of the physical parameters of the optoelectronic transducer, the motion scene, or the presence of off-line blackbody sources. The ability of the proposed method to reduce FPN is measured by evaluating the structural similarity (SSIM) index employing a blackbody-based FPN reduction technique as a reference. This new interpretation of the FPN opens avenues to create new cameras for low-energy photons with the ability to perform denoising by digital refocusing.

## Introduction

Researchers in the field of digital imaging technology are constantly attempting to improve image resolution, whether in spatial, spectral or temporal quality, particularly because such capabilities are required to discover new information in fields such as astronomy, medicine, and biology, among many others. Imaging technologies present distinct types of noise characteristics, varying in intensity, features, and nature. For example, the noise characteristics of various infrared (IR) imaging systems vary in intensity and spatial and temporal features depending on the nature of the IR-radiation transducer and the electronics of the readout integrated circuit (ROIC). These noise characteristics become superimposed on the captured IR radiation, thus causing image degradation, which can be observed by naked-eye visual inspection when viewing a raw digital image, since the resulting images exhibit a signal-to-noise ratio lower than 1. The numerous varieties of noise that affect imaging systems^1–4^, can be classified based on their temporal characteristics. Some noise sources vary as rapidly in time as the imaging system integration time. In contrast, other noise sources remain fixed for many integration time periods, forming a static pattern over the scene, as if one were viewing the image through a dirty or streaked window. This latter type of noise is typically called fixed-pattern noise (FPN) and is attributed to manufacturing imperfections, inhomogeneous pixel responsivities, dark currents of detectors, and typical readout architectures, such as a passive-pixel sensor or active-pixel sensor, introducing both temporal and spatially FPN^1,3^. In general terms, non-uniformity correction techniques can be classified into two main families: calibration-based and scene-based techniques^5^. The calibration method uses an absolute temperature reference; for this purpose, we used a blackbody radiation source, and for quantitative estimation of quality assessment, we used the blackbody radiation two-point (BBRTP) calibration method. Scientific applications require the highest radiometric precision available; thus, bulky and expensive calibration sources must be employed frame-by-frame to reduce FPN. In contrast, military applications and certain industrial applications require on-line classification capabilities; in this case, transducer technology, ROIC technology, and application dependency model-based algorithms must be specially designed and tuned to filter out the FPN on a case-by-case basis.

Here, we present technology- and application-independent solutions to the problem of FPN in the context of wide-band IR imaging systems. However, the proposed solution can also be tested in visible-IR-hyperspectral and terahertz imaging systems. The key idea is to realize that FPN behaves as an optical object located at an image depth of infinity. When a collection of images in the same scene is captured with relative displacement between them, such as a scene image light field, such set can be used to achieve digital refocusing to any nearby objects in the scene. Naturally, this approach filters out the FPN as a consequence of the defocusing. This denoising is based on the most notable property of this type of noise, namely, that its location is fixed on the images over time.

**Figure 1:**
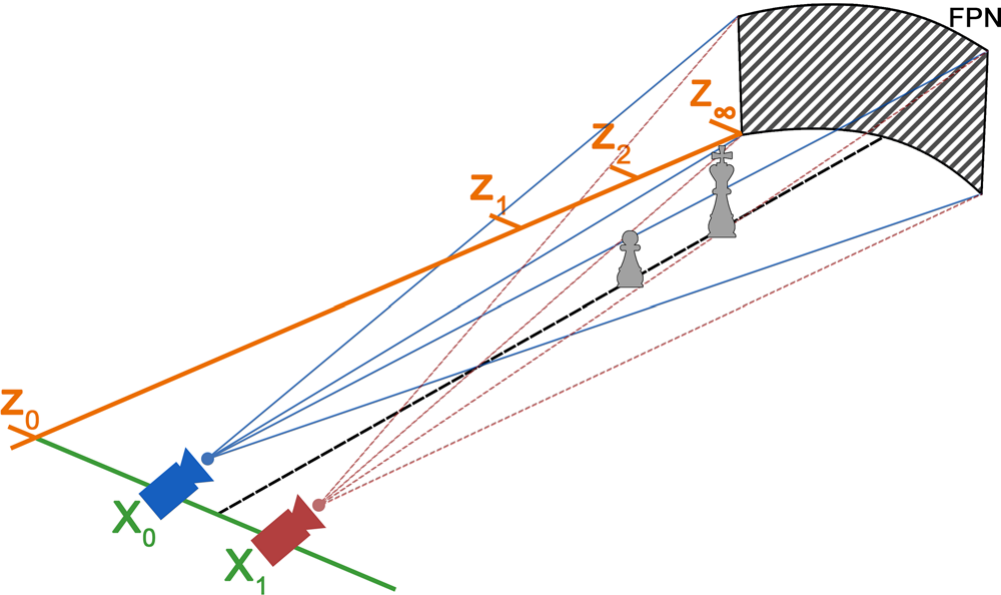
Schematic of the FPN as an object at a depth of infinity, *Z*_∞_. The imaging system superimposes the same noise pattern on the captured images of the scene when located at the spatial positions (Z_0_, *X*_0_) and (Z_0_, *X*_1_). Thus, regarding the relative position of the objects in the scene, the FPN appears as an object at a depth of infinity. This property of the FPN creates a parallax effect when observing close objects (e.g., objects at Z_1_ and Z_2_) from the positions *X*_0_ and *X*_1_.

To explain this new concept, we show in Fig. 1 a simplified scene where FPN is depicted as an optical object located at the depth infinity. The Z coordinate describes the scene depth. The baseline of the camera is at the position Z_0_, the plane of nearby objects is at the position Z_1_, intermediate objects are located at the position Z_2_, and the FPN at *Z*_∞_ represents the optical infinity of the system.

This new way of treating the FPN as an optical object at a depth of infinity allows one to develop a global solution to the problem of FPN reduction. Specifically, in this work, we build a plenoptic system with an IR camera with high FPN content using scanning. We show that it is possible to perform denoising by defocusing the FPN. For this purpose, the light field *L*_*f*_ of the scene (plenoptic collection of images that captures scene information as a 4-dimensional function) is processed using a digital refocused algorithm^6–11^. In this work, we decided to employ the seminal work developed by Ren Ng^6^ known as the Fourier slice photography theorem. As a result of performing digital refocus, the effect of the FPN on objects at planes close to the imaging system is significantly mitigated. The level of noise reduction is quantified by the structural similarity (SSIM) index^12^, showing that it is possible to obtain a high degree of FPN cancellation using as a reference a blackbody-based FPN calibration technique.

## Results

The reconstruction of digitally refocused images at far object planes shows severe FPN. However, we shall show that FPN is continuously reduced when refocused images at closer object planes are reconstructed by applying the refocusing algorithm to the captured light field.

**Figure 2:**
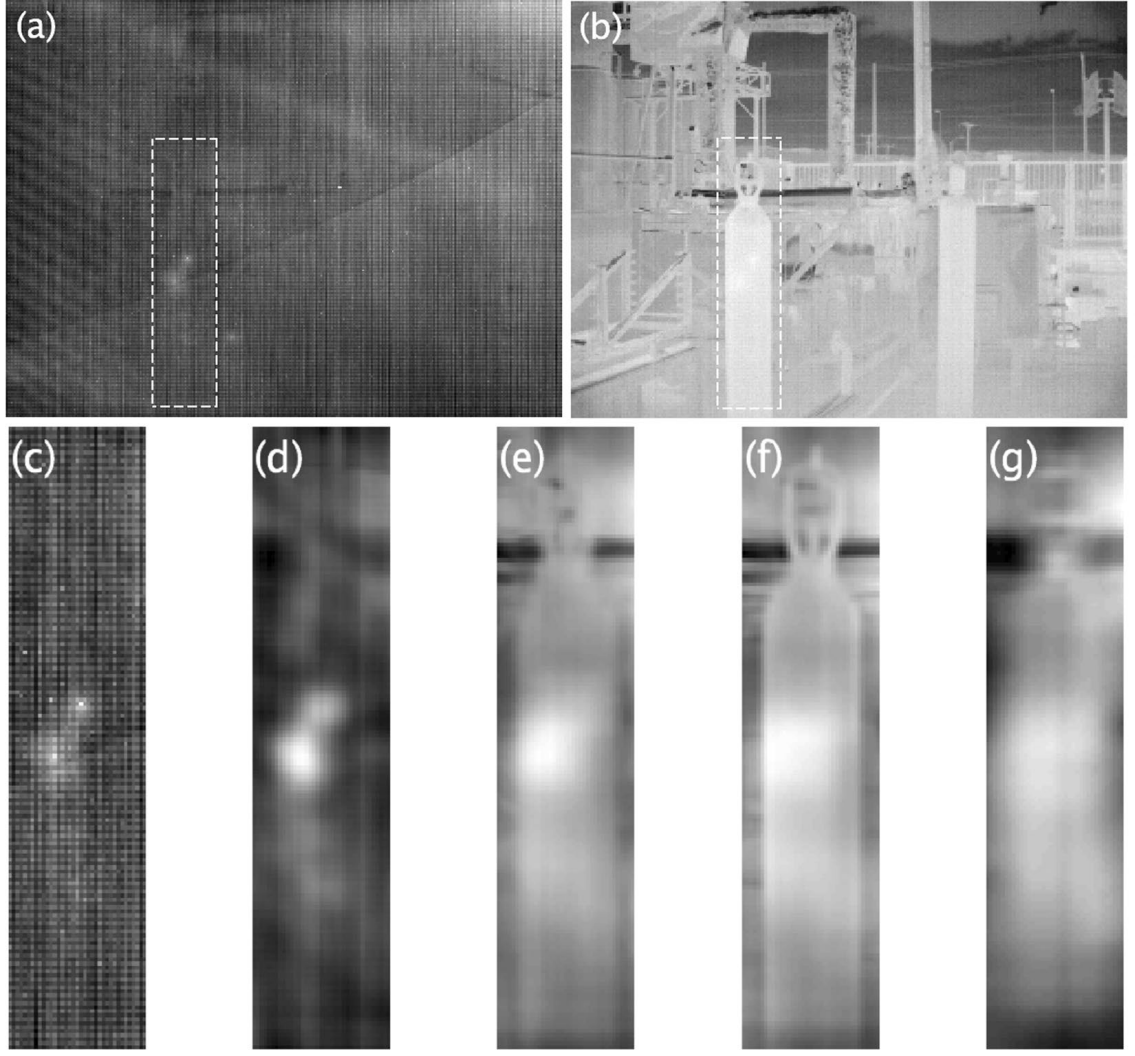
FPN reduction attained by the proposed method for the Cedip IR camera in an industrial scene. (**a**) Raw image and region of interest (RoI) with a high presence of FPN, which lies inside the segmented-lines rectangle and it corresponds to an industrial gas cylinder. (**b**) The scene image and RoI for the nearest scene objects corrected by the BBRTP method. (**c**–**g**) RoI reconstructed using the proposed method at the focal planes: (**c**) *N* = 1, (**d**) *N* = 70, (**e**) *N* = 140, (**f**) *N* = 174, and (**g**) *N* = 240 for the closest object.

The image of Fig. 2a shows the scene that was captured by the plenoptic IR system (using a Cedip camera and scanning to produce a matrix of 16 × 16 images with a step of 15 mm), which corresponds to an industrial sector with slow temperature variations with high thermal contrast contents. The Cedip camera is a JADE-UC uncooled microbolometer focal-plane-array (FPA) model that operates in the long-wave IR range of 8 to 12 μm and is equipped with a 14-bits, 320 × 240 FPA.

The image of Fig. 2a was FPN corrected by using the two-point calibration method (TPC)^13–15^, that utilizes a first-order model for the camera response. This calibration technique was applied using two uniform radiation sources acquired from two high-accuracy blackbody radiators in the Mikron M300 series, at 20 °C and 30 °C, respectively.

The region of interest (RoI) in Fig. 2a is shown as a rectangle of segmented lines. This RoI corresponds to a gas cylinder located 3 m from the camera (closest object in the image scene) and is used to enable a comparison of results for this entire work. It is important to note that we have used the most complex camera pixel region to locate our object of study since there are two bright spot artifacts in the image (attributed to partially saturated pixels of the FPA).

Figure 2b shows the same image of Fig. 2a without FPN correction; this is a raw image, and corresponds to an image of the *L*_*f*_ with severe FPN. From left to right, Fig. 2c shows a sequence of 5 RoI images corresponding to the refocusing planes, namely, c) *N* = 1, d) *N* = 70, e) *N* = 140, f) *N* = 174 (the exact refocused image), and g) *N* = 240, thereby suppressing FPN. The reduction of noise by defocusing is limited by the nearest refocused object plane. In other words, we must determine the *N* associated with the plane of the focused near object. For this case, the value is *N* = 174, and it is obtained using the level of focus algorithm based on the Brenner function^16,17^, which coincides with naked-eye visual observations.

The degree of denoising FPN was quantified by SSIM index^12^, which employs an image reference free of FPN and the corresponding image at a refocused image from the raw-data light field. The range of this index are values from 0 to 1, where the lowest value (SSIM = 0) corresponds to entirely different images and the highest value (SSIM = 1) corresponds to identical images. Thus, we used the BBRTP calibration method, which allows generating the reference sets of light fields when applying the method to the raw-data light field. When applying the plenoptic algorithm for quantitative comparison, the refocused images generated from the raw-data light field are always compared with the refocused images from the BBRTP light field; thus, the SSIM index is a distance measure between these refocused images.

We studied the level of FPN reduction on the RoI by digital refocusing at 300 different object planes. As a reference, we used the same RoI images corrected using the BBRTP method. Since the images being compared differ only in the amount of FPN, a high value of the SSIM index is directly associated with a high level of noise reduction. We emphasize based on our experience that values greater than 0.4 correspond to levels of noise reduction that make the residual FPN imperceptible in naked-eye inspection. Additionally, an SSIM index greater than 0.4 means that the FPN has been reduced to an extent to which objects can be distinguished in the image. Note that in IR imaging artefacts such as dead, saturated, and flickering pixels and any computational imaging processing may change the IR contrast of the raw scene imaging. Thus, the SSIM index primarily corresponds to the SSIM of the objects within the IR image.

**Figure 3:**
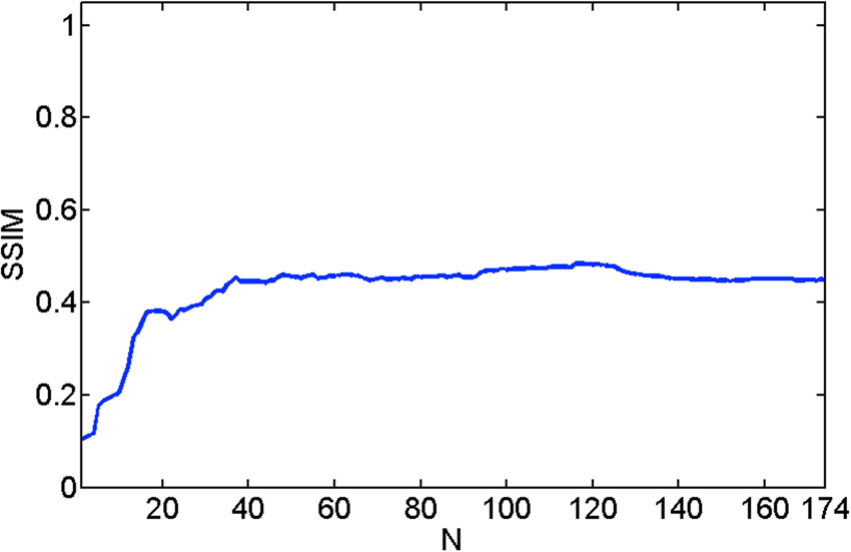
Dependence of the spatial SSIM index versus the dimensionless parameter *N*, which represents the different focal planes.

The graph in Fig. 3 shows the dependence of the SSIM index versus the refocusing parameter, *N*, of the nearby object presented in the RoI. The first values of *N* correspond to distant planes, where there is a high presence of FPN, as evidenced by reaching SSIM values lower than 0.13. As the closest planes are refocused (corresponding to large values of N), the FPN begins to defocus. This result is most strongly pronounced in the first 100 refocused planes, where the SSIM index increases from 0.13 to 0.49. Although there is a tendency to continue improving the FPN reduction, we can use the focused image only up to the value of *N* = 174.

**Figure 4:**
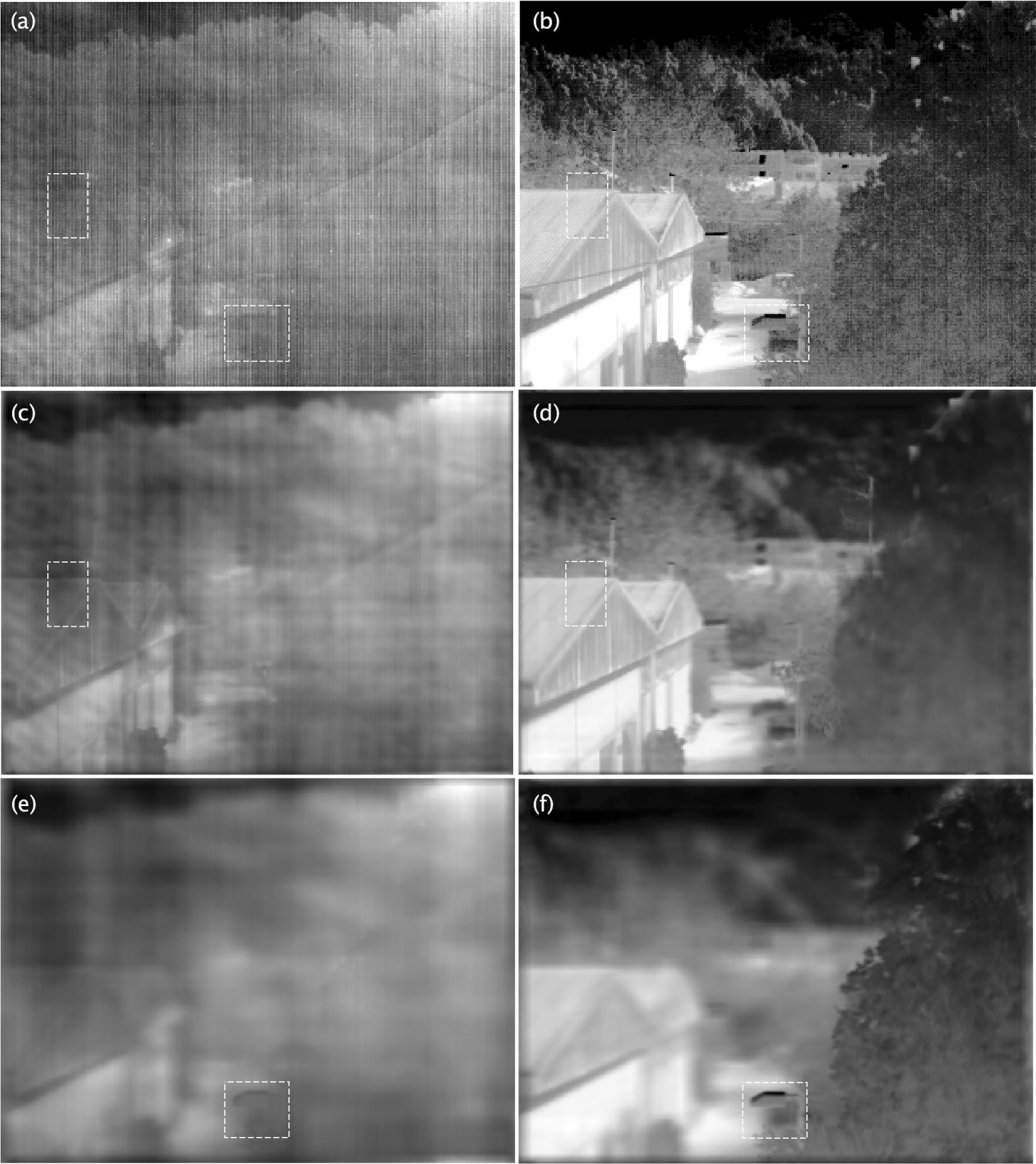
Winter season scene in which only half of the scene is under direct sunlight illumination. (**a**) Raw image from the light field, where two RoI are highlighted with rectangles. (**b**) Image from the light field obtained by applying the BBRTP calibration method to the raw-data. (**c** and **e**) Shows refocused object planes with *N* = 47 (roof of a near house, SSIM  = 0.55) and *N* = 100 (public luminary, SSIM  = 0.54), respectively. (**d** and **f**) Are the same as (**c** and **e**), respectively, for the BBRTP-calibrated images.

**Figure 5:**
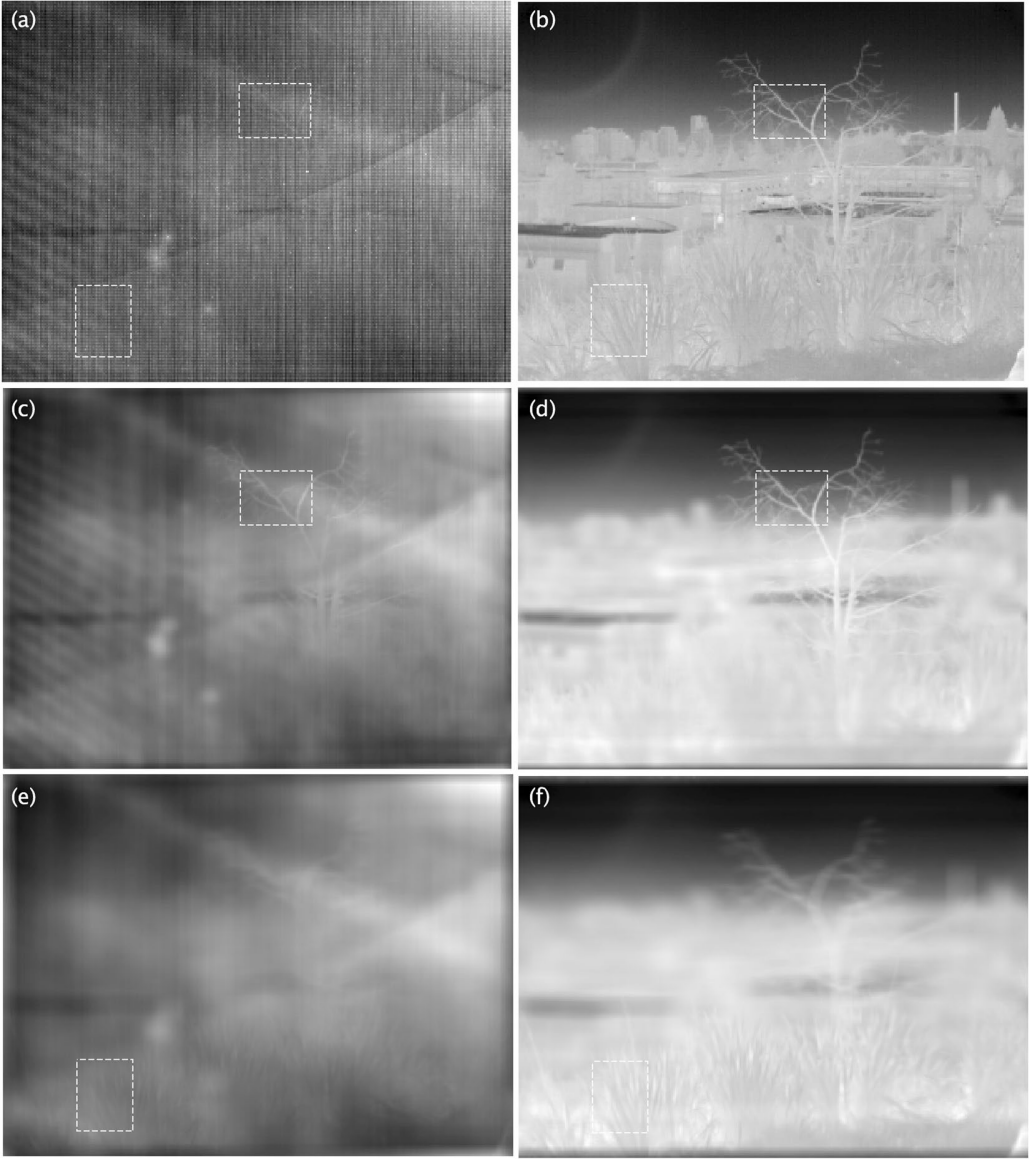
Daylight city-landscape scene in the spring. (**a**) Raw image from the light field, where the RoI are highlighted with rectangles. (**b**) Image from the light field obtained by applying the BBRTP calibration method to the raw-data. (**c**) and (**e**) show refocused object planes with *N* = 70 (tree, SSIM  = 0.65) and *N* = 117 (shrubbery, SSIM  = 0.77), respectively. (**d**) and (**f**) are the same as (**c**) and (**e**), respectively, for the BBRTP-calibrated images.

To experimentally demonstrate that the method works satisfactorily in different scenarios, we apply the proposed method to two additional scenes. The first scene, shown in Fig. 4, corresponds to a winter scene where only half of the scene is under direct sunlight illumination. The second scene, shown in Fig. 5, corresponds to a daylight city-landscape in the spring. In Fig. 4, we selected two regions of interest, a portion of the roof of a warehouse and a street light, which are shown in Fig. 4c,e at refocused object planes with *N* = 47 and *N* = 100, respectively. In both cases, the structures can be recognized within the RoI, which does not occur in the raw-data in Fig. 4a. Similarly, in Fig. 5 we selected two regions of interest, a portion of the tree and a shrubbery, which are shown in Fig. 5c,e at refocused object planes with *N* = 70 and *N* = 117, respectively. Again, in both cases, the structures can be recognized in the RoI, which does not occur in the raw-data in Fig. 5a. The entire datasets for the above described light fields are publicly available, both raw data and BBRTP calibrated data^18^.

The results show that the proposed noise cancellation method reaches significant levels of FPN reduction. Such FPN reductions are quantified by SSIM index reaching values corresponding to a human naked eye’s structure recognition. Of course, the SSIM index difference with respect to the blackbody FPN method is not attributable to the structural objects scene recovering process, but to the contrast modification introduced by both techniques to the raw-data in the process of the FPN correction. Notably, unlike standard techniques^19–23^, our method does not require knowing physical parameters associated with the optoelectronic transducer system with which the images are acquired, the motion scene, or the presence of off-line blackbodies for estimation of non-uniformity sensor parameters.

**Figure 6:**
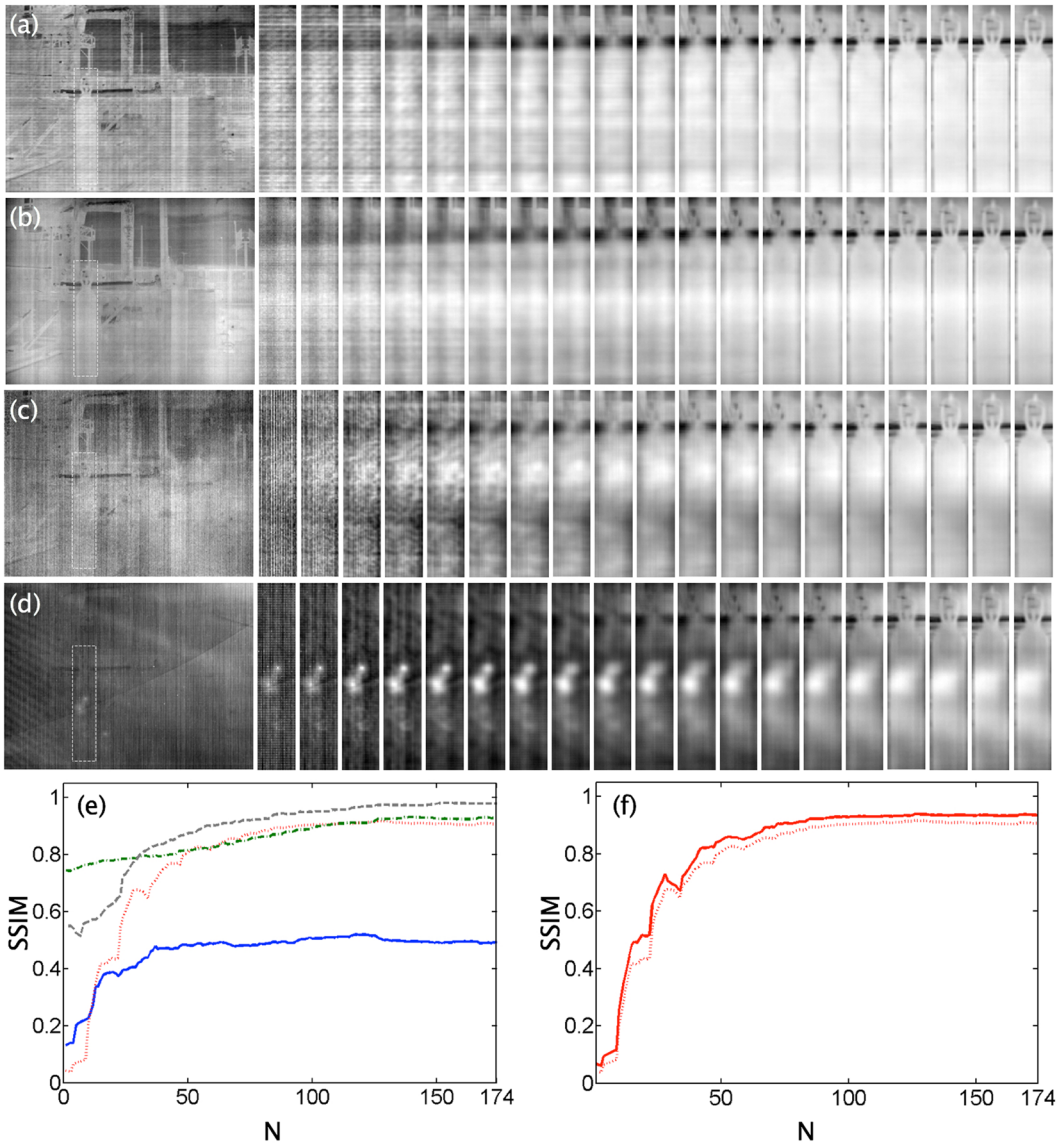
Method evaluation for different scenarios. Set of 19 object planes for various IR transducer technologies, from top to bottom: (**a**) Amber, (**b**) Lumasense, (**c**) QDIP, and (**d**) Cedip IR cameras. (**e**) SSIM index versus refocusing planes: Amber, dashed-line (grey); Lumanesense, dashed-dotted line (green); QDIP, dotted line (red); and Cedip, solid line (blue). (**f**) SSIM index for QDIP IR technology for two different *L*_*f*_ architectures, namely, an *L*_*f*_ formed with a single camera scanning (dotted line) and a *L*_*f*_ formed with a set of *m* × *n* cameras (solid line).

Evaluating the effectiveness of the proposed method for reducing the FPN on different IR transducer technologies, where each type of transducer presents FPN with distinctive features, is quite interesting. Using pre-recorded blackbody data and the previously used *L*_*f*_ corrected by BBRTP, it is possible to artificially generate an *L*_*f*_ containing real FPN from the blackbody data. For this experiment, three additional cameras from different technologies are considered. The first camera is a commercial Amber camera, model AE-4128, which operates in the mid-wave infrared (MWIR) (3–5 μm) region and is equipped with a cooled InSb FPA with 128 × 128 pixels and 16-bits resolution. The second one is a commercial Lumasense MWIR camera, model MC320MHT, which operates in the range of 3–5 μm and uses a 16-bit uncooled FPA of 320 × 240 pixels. The third camera is a quantum-dots infrared photodetector (QDIP) camera, which operates in the mid- and long-wave IR regions (3–5 μm and 8–12 μm), is equipped with an InAs QDs/InGaAs/GaAs cooled FPA of 356 × 240 pixels, and quantizes data in 14-bits. For this experiment, blackbody samples of each camera at two different temperatures were used. These samples were taken without the use of built-in FPN compensation options. Specifically, blackbody samples were recorded by the Amber camera at temperatures of 20 °C and 30 °C, by the Lumasense camera at 100 °C and 350 °C, and by the QDIP camera at 26 °C and 50 °C. By employing these data and a TPC version of the *L*_*f*_, the noise pattern of these technologies is artificially superimposed on the clean *L*_*f*_ using a first-order approximation for the response of the camera^13–15^, Subsequently, the *L*_*f*_ with FPN is generated, and the proposed method is applied (see Fig. 6).

From top to bottom, Fig. 6a shows the outcome of refocusing the RoI for the Amber, Lumasense, QDIP and Cedip Technologies. The first image of each row corresponds to an image of the *L*_*f*_ with the noise of each transducer technology, and the images to the right show the RoI at different refocusing planes that are separated from each other by a Δ*N* = 10. The last image corresponds to the best-refocused plane, which is for *N* = 174. In all of the cases, we considered the use of a single camera when generating the corresponding *L*_*f*_; namely, for each IR technology, a unique noise pattern was rendered on all the *L*_*f*_ images. Note that the Cedip thermal transducer generates the strongest FPN. Indeed, for this case, the scene object cannot be distinguished from the noise. Figure 6b shows the SSIM index versus digital refocused planes for the four analyzed technologies. In all the cases, the object in the RoI is focused at the plane *N* = 174, and for all the photonic transducers, the SSIM is higher than 0.8 at that object plane. For the Cedip thermal transducer technology, the $SSIM=0.49$ at *N* = 174. Note that an SSIM index value greater 0.4 means that objects can be distinguished in the image. Among all the technologies, the largest reduction of FPN is achieved for the photonic transducer QDIP camera, which begins with an index of structural similarity $SSIM=0.04$ and later reaches a value of $SSIM=0.90$ (close planes). The greatest FPN reduction is achieved for the photonic transducer Amber technology with a $SSIM=0.98$.

Furthermore, to show the robustness of the proposed method, we conducted three complementary performance evaluations. The first one considers another way to record an IR *L*_*f*_. In Fig. 6c, we plot the variation of the spatial SSIM index for two different architectures when generating the *L*_*f*_ for the QDIP technology (with photonic transducers). The first architecture is a single camera and a scanning system, and therefore, a single noise pattern is replicated in all the images of the *L*_*f*_ (dotted line). In contrast, the second architecture considers a different camera of the same technology for each image of the *L*_*f*_ (solid line). This design is simulated by using the procedure^15^ for synthesizing different FPN samples that preserve the technology-dependent spatial structure of the real FPN of a camera. As shown in Fig. 6c, the method performs equally well under both types of FPA architectures for generating the corresponding *L*_*f*_. This result provides clear evidence of the robustness of the proposed method under different *L*_*f*_ architectures.

The second and third complementary performance evaluations were conducted using the Cedip camera and scanning. To study how reducing the *L*_*f*_ size affects FPN cancellation, three cases are presented in the second evaluation. Specifically, a light field of 16 × 16 images was reduced to 8 × 8 and 4 × 4 images while maintaining a step between adjacent images of 15 mm.

Table 1 indicates that a reduction of the *L*_*f*_ size produces a reduction of the SSIM index from $SSIM=0.49$ to $SSIM=0.47$. Regarding FPN reduction performance, we see that a smaller *L*_*f*_ shows little diversity of spatial frequencies and generates refocused images with greater depth, which makes smaller *L*_*f*_ relatively limited in regards to forming a discrete plane of refocused elements. This constraint does not allow a precise selection of information; therefore, the residual noise of the FPN structure remains even when one focuses only on the object of interest located in a near optical plane. However, in all cases, the SSIM index is similar to the ones computed for an *L*_*f*_ of 16 × 16 images. Note that this phenomenon occurs even in the event of the smallest *L*_*f*_ that contains only 4 × 4 images.

**Table 1:** SSIM index for *L*_*f*_ formed with a different number of images.

*m* × *n*	Δ*x* [mm]	*N*	SSIM
4 × 4	15	43	0.47
8 × 8	15	94	0.47
16 × 16	15	174	0.49

In the third case, the performance of the method is studied on 16 × 16-image light fields with a different step size between adjacent images. Three different *L*_*f*_ with steps of 15 mm, 10 mm and 5 mm were experimentally acquired. The results of applying the proposed method to each one of these light fields are shown in Table 2.

**Table 2:** SSIM index for different spatial separation between *L*_*f*_ consecutive images.

*m* × *n*	Δ*x* [mm]	*N*	SSIM
16 × 16	5	63	0.51
16 × 16	10	118	0.48
16 × 16	15	174	0.49

As shown in Table 2, by reducing the separation between images, the number of planes between optical infinity and the best-refocused plane for the closest object decreases in number, from 174 planes to only 63. This decrease coarsens the depth of field at each discrete plane, which limits the refocusing precision on specific objects, although the SSIM index is not affected significantly, and the quality of the images is maintained.

## Discussion

We have proposed a new technology-transducer and application-independent method for FPN reduction that does not rely on the use of physical information of the FPA or on using external reference scenes (such as calibrated blackbody sources). In particular, we have experimentally demonstrated the effectiveness of the proposed method in the case of a scanning plenoptic IR system with a single camera. The method was tested by varying the size of the captured *L*_*f*_ and varying the displacement between consecutive images in the *L*_*f*_. These tests allow one to appreciate that noise reduction is robust under both variations of the experimental configuration. Notably, a small *L*_*f*_ of 4 × 4 images produces a high increase of the SSIM index, which makes this method very promising for future IR imaging and a visible-IR-hyperspectral imaging system with *L*_*f*_ ability.

Furthermore, we have demonstrated the robustness of the method for three other IR transducer technologies, using digital addition of real FPN characteristics from each technology in our *L*_*f*_ free of FPN. In all cases, we obtained high noise cancellation, which strongly suggests that the method is technology independent. For studied technologies, the most notable FPN reduction occurs for the QDIP camera, for which the SSIM concerning the FPN free reference increases by 0.86. We also performed simulations of plenoptic *L*_*f*_ obtained with an array of IR cameras, where each one of the *L*_*f*_ images was captured by an independent camera. Here, each image has different FPN structures, and the remarkable property of the method is that even in such cases, the noise reduction is efficient and comparable to the case of using a single camera and scanning system for capturing the *L*_*f*_ (same FPN for all the images).

In the next few years, the proposed method is expected to have technological implications in the development of new cameras, for low-energy photons, such as IR and terahertz cameras, and for visible and IR spectral selected photons (hyperspectral cameras) with the ability to perform denoising by digital refocusing.

## Methods

**Figure 7:**
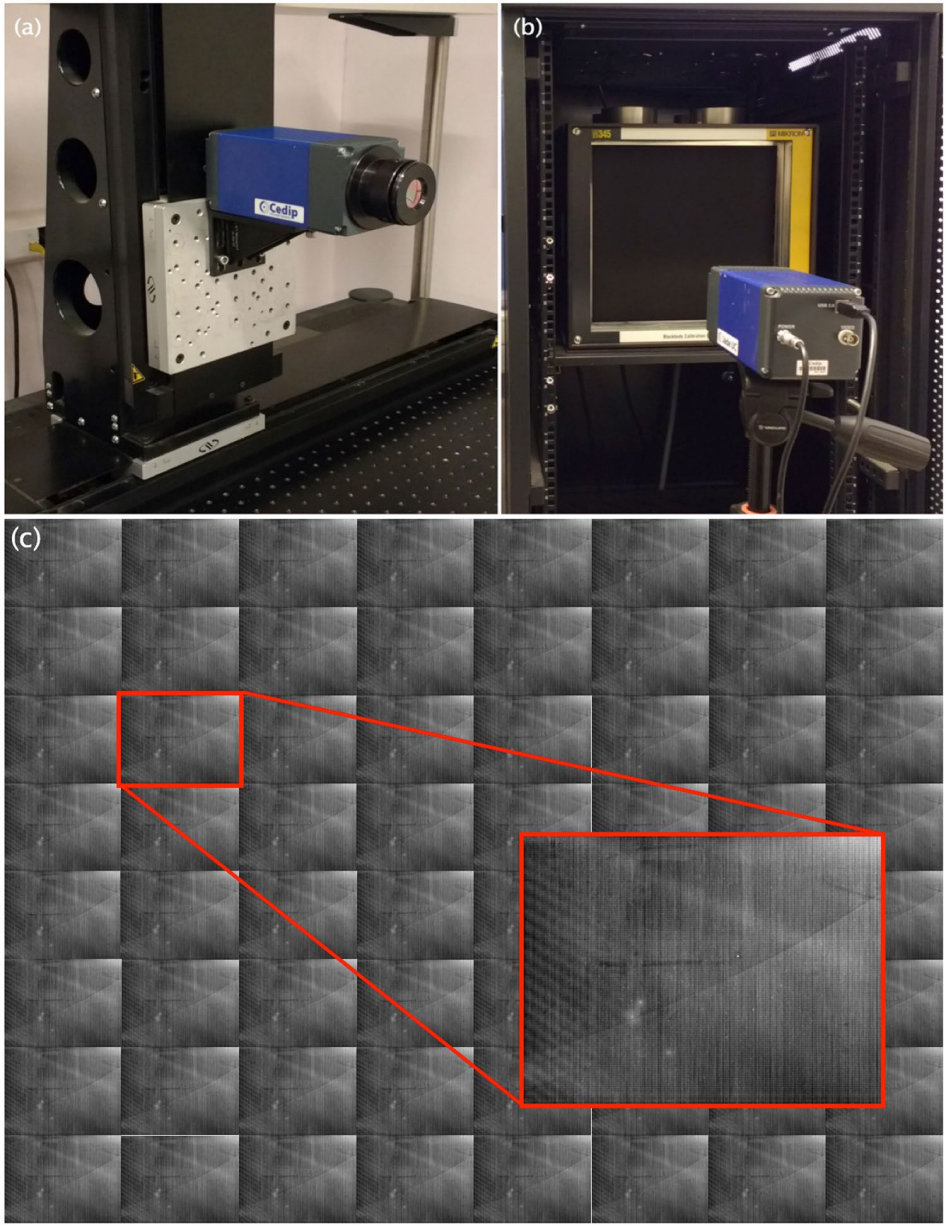
(**a**) Experimental configuration for the acquisition of the individual images at the light field using transversal scanning, where the IR Cedip camera has been installed in a high precision positioning system. (**b**) Experimental configuration for capturing raw images from a uniform scene from a blackbody-radiation-calibrated source Mikron 345. Two images are registered with the blackbody radiation source at 20 °C and 30 °C. (**c**) Light field with the presence of FPN on an 8 × 8-images subset of the entire *L*_*f*_ of 16 × 16-images.

One method to acquire a scene *L*_*f*_ is through a two-dimensional transverse scanning employing a single camera, see Fig. 7a. This *L*_*f*_ capture procedure was implemented with the following equipment: an optical bench, a high-precision linear translation stage with a displacement range of 600 mm with DC motor and rotary encoder (model: IMS600CC, Newport), a high-precision vertical translation stage (minimal translation of 1.25 μm) with a displacement range of 300 mm and with a DC motor (IMS-V Series, model: IMS300V, Newport) mounted on a right angle bracket (model: EQ120, Newport), and a 2-axis universal driver with an Ethernet connection (model: XPS-Q2, Newport) for controlling of vertical and horizontal translation stages. The digital camera was a Jade-UC, Cedip Inc., which integrates the incident radiance using an uncooled microbolometer FPA sensor of 320 × 240 pixels that operates in the range of 8–12 μm and has a dynamic range of 14-bits. This camera was operated without using its built-in FPN compensation options (raw-data imaging), and the formation of the image on the sensor used a focal length lens of *f* = 24 mm and an *F*-number of *F* = 1.1.

Through the overall equipment arrangement and operation, as shown in Fig. 7, the light field ${L}_{f}(x,y;m,n)$ was generated. The coordinates (*x*, *y*) correspond to the image pixels, and the coordinates (*m*, *n*) correspond to the position of each *L*_*f*_ image. In our case, the images were of 320 × 240 pixels in an arrangement of 16 × 16 images. The offset between (*m*, *n*) coordinates of the *L*_*f*_ was 15 mm for the vertical and horizontal directions.

The digital refocusing was accomplished by means of the Fourier slice photography theorem^6^:

1 $${{\mathscr{P}}}_{\alpha }[{L}_{f}]={ {\mathcal F} }^{-2}\circ {{\mathfrak{B}}}_{\alpha }\circ { {\mathcal F} }^{4}[{L}_{f}],$$

Using the digital refocusing operator, ${{\mathscr{P}}}_{\alpha }$, it is possible to select a scene’s spatial frequency information from *L*_*f*_ to reconstruct images focused on different object planes determined by the α parameter, where small α values correspond to far object planes. Thus, an effective refocused image on a fixed object plane is obtained through the two-dimensional inverse Fourier transform, ${ {\mathcal F} }^{(-\mathrm{2)}}$, from a two-dimensional slice information, ${{\mathfrak{B}}}_{\alpha }$, from the Fourier transform in four-dimensions, ${ {\mathcal F} }^{\mathrm{(4)}}$. For this particular work, the α values are related to different focal planes obtained in such a way that every $N=\mathrm{1,\; 2,\; 3,}\cdots$ is associated with ${\alpha }_{1}=\mathrm{0.999,}{\alpha }_{2}=\mathrm{0.998,}{\alpha }_{3}=0.997\ldots ,{\alpha }_{N}=1-0.001\times N$, which take values equally spaced between them. Finally, 300 effective refocused images were reconstructed and arranged from the scene image farthest object plane to the closest object plane, i.e., from *N* = 1 to *N* = 300.

To quantify the quality assessment of the proposed method for denoising FPN, we used the SSIM index^12^, which allows one to quantify the SSIM between a reference image and a distorted image by considering their luminance, contrast, and structure by considering that these three parameters are relatively independent. This index has been developed for the visible region of the electromagnetic spectrum by considering that human perception is adapted to extract structural information of scenes. In our case, as reference images in a particular refocused plane, we used those refocused images obtained from the BBRTP corrected light field, and the distorted images are refocused images from the raw-data light field.
